# Pseudoaldosteronism due to mutation of SCNN1A gene: a case report

**DOI:** 10.1186/1687-9856-2015-S1-P127

**Published:** 2015-04-28

**Authors:** Can Thi Bich Ngoc, Vu Chi Dung, Bui Phuong Thao, Nguyen Ngoc Khanh, Maria-Christina Zennaro, Stefan A Wudy

**Affiliations:** 1 Department of Endocrinology, Metabolism & Genetics; National Hospital of Pediatrics, Hanoi, Vietnam; 2 Departement de Genetique; Hopital Europeen-Pompidou, Paris, France; 3 Pediatric Endocrinology & Diabetology; Center of Child and Adolescent Medicine; Justus Liebig University, Giessen, Germany

## Introduction

Pseudohypoaldosteronism type 1 (PHA1) is a rare inherited disease characterized by resistance to the actions of aldosterone. It was first described in 1958 by Cheek and Perry, and common clinical manifestations include salt wasting, hyperkalaemia, metabolic acidosis and elevated plasma aldosterone levels in the neonatal period.

## Objective

To describe clinical characteristics, laboratory features and management of one Vietnamese patient with pseudohypoaldosteron.

## Subject and methods

Clinical features, biochemical finding, mutation analysis and management in a 1 months-old-boy was studied. Based on analysis of this patient’s clinical symptoms associated with biochemical examination, the urinary steroid metabolomics analysis was performed using gas chromatography spectrometry and mutation analysis of SCNN1A was performed using PCR & direct sequencing.

## Results

Patient is the first child normal delivery with the gestation age of 41 weeks, birth weight of 3200 g, and onset of the disease at 7 days of age. He presented with lost weight, dehydration without vomit, diarrhea or hyperpigmentation. He was admitted with the features of cyanosis, allorhythmic, electrolyte imbalance with sodium of 119 mmol/l, potassium of 7.4 mmol/l. Investigation show pH 7.26, PCO2 34 mmHg, PO2 110 mmHg, HCO^-^_3_ 18mmol/l, BE -10, plasma 17OHP level: 2,4 ng/ml, testosterone level: 1.94 nmol/l, Cortisol 8am: 2662,8 pmol/l, Ure 7.4 mmol/l, Creatinine 44.2 umol/l, Glucose 4.8 mmol/l. the urine steroid metabolomics analysis showed extensive excretion of aldosterone ID-ISTD1 of 1157.41 µg/l. Novel homozygous mutation (c.1668C>A; p.S556R) of SCNN1A gene was identified in the proband. He was treated with florinef of 0.1 mg/kg/day for electrolyte balance. He had complication of intestinal perforation and died due to infection. In conclusions, PHA1 causes severe hyponatremia, metabolic acidosis, and life-threatening hyperkalemia, with normal 17-a-hydroxyprogesterone levels and high excretion of aldosterone levels

Written informed consent was obtained from the patient's parent or guardian for publication of this Case report (and any accompanying images). A copy of the written consent is available for review by the Editor-in-Chief of this journal.

